# Complex Problems and Dealing with them on a Research Methods Course in a Business School

**DOI:** 10.1007/s11213-022-09624-w

**Published:** 2022-11-17

**Authors:** Stephen Harwood

**Affiliations:** University of Edinburgh Business School, 29 Buccleuch Place, EH8 9JS Edinburgh, UK

**Keywords:** Problem structuring, Research methods, Pedagogy, VIPLAN Methodology, Cybernetics, Systems thinking

## Abstract

This study offers a conceptual explanation of a holistic methodology that has utility in how we engage with complex situations. This is the VIPLAN Methodology developed by Raul Espejo and first published in 1988. A case is presented that evaluates whether this methodology has impact when tacitly embedded in a postgraduate research methods course. The underlying argument is that research can be usefully viewed as a form of engagement in complex problem situations, with one challenge being to make sense of situational complexity and establish the question. Thus, it becomes appropriate to introduce the principles underpinning this methodology into a research methods course in order to develop student understanding of how to move from making sense of the situation to a clearly defined problem that is then handled. The study examines a research methods course delivered annually over a period of three years, but drawing upon data from the previous two years, permitting a five year perspective. The evidence from this study in the form of marks attained and proposal - dissertation topics, indicates that this more holistic approach to a research methods course has a positive impact, especially in terms of establishing a research question. It is concluded that the introduction of the principles underpinning this methodology into a research methods course does enhance the student ability to handle complex situations.

## Introduction

The complexity, uncertainty and turbulence of the situations confronting organisations often leaves managers unclear about what to do. Thus, the ability to handle these ‘complex problems’ has been repeatedly identified by the World Economic Forum (WEF 2016, 2018, 2020) as one of the key skills required for future employability. A research methods course offers the opportunity to develop this skill. Moreover, this is grounded in the view that students need not be future producers of research. Rather, that they are likely to be future consumers of research, as well as future active participants in problem-solving (Good 1936; Schutt et al. 1984; Hardcastle and Bisman 2003). Thus, a research methods course can have relevance for later employment by developing their understanding of the challenges of inquiry: “For these students, the research methods course can improve general skills of inquiry” (Schutt et al. 1984: 235–236). An immediate benefit is the application to help students make sense of interests and establish their research focus for a dissertation, which can present a major challenge to students and be a potential source of anxiety.

This paper explores the notion of a complex problem, how to handle it and the impact of embedding an appreciation of how to handle complex problems into a postgraduate research methods course.

## A Need to Handle Complex Social Situations

A complex problem arises in situations defined as characterised by complexity, uncertainty and turbulence, such as associated with Covid 19 (VSO 2021) and Climate Change (IPCC 2021). These situations have been referred to as wicked (Churchman 1967, Rittel and Webber 1973), messy (Ackoff 1974; Eden et al. 1983) and fuzzy (Zadeh 1965; Bellman and Zadeh 1970).

Messy problems are faced by many organisations. For example, the development of a marketing strategy will require sense-making of possible futures. Likewise, hybrid working due to Covid 19, has created new expectations about longer term working practices. The danger with complex situations is captured by Bhardwaj, Crocker, Sims and Wang (2018) who argue that managers not uncommonly fail to understand what is the problem:

Jumping to conclusions, imposing solutions, limiting information search and alternative generation in a preference for action, taking shortcuts under time pressure, and paying little attention to those affected were among the reasons for failure (Bhardwaj et al. 2018: 279).

The consequences are bad decisions, which Nutt (1999) attributes to lack of attention to understanding the situation and hence establishing what the problem really is. In other words, the approach to dealing with complex situations is flawed.

The challenge of understanding how to appropriately make sense of complex situations may stem back to university courses and the way we teach. Eden (1987) suggests that university courses in the West tend to focus upon solutions and not the problem. The emphasis is upon well-structured problems with right answers that do not involve negotiation, emotion or tension (Eden 1987).

This suggests that more attention is given in university courses to how to formulate the problem. Indeed, Bhardwaj et al. (2018) call for approaches for structuring and framing problems to be embedded into strategic management courses to highlight the problematic nature of strategizing. This can also apply to research methods courses. Strangman and Knowles (2012) argue that research methods courses tend to emphasise problem solving rather than problem formulation, despite formulation being recognised as part of the research process (e.g. Onwuegbuzie and Leech 2005; Hesse-Biber 2015).

Approaches to problem formulation, particularly for complex situations, exist in the form of ‘problem structuring methods’ (PSMs) (Rosenhead 1989), such as Soft Systems Methodology (Checkland 1981), Strategic Choice Approach (Friend and Hickling 1987), Strategic Options Development and Analysis (Eden 1988) and the VIPLAN Methodology (Espejo 1988, 1992). Further, Harwood (2019) makes a general call for the embedding of PSMs, and specifically the VIPLAN Methodology, within the university curriculum to develop problem formulation skills.

Nevertheless, it is unclear whether a PSM has any impact upon the student learning experience and specifically the development of the capability to problematise a situation and offer solutions. A research methods course is an example where the development of this capability might be expected, since this course can be the precursor to a dissertation project whereby students need to establish a research question and then design and implement a study to address the question. Indeed, Harwood (2016) proposes that research methods courses can be designed to embed problem structuring into them.

This suggests a research gap. It has not been ascertained whether Harwood’s (2016) proposal of embedding PSMs in a research methods course would result in better student problematisation capability.

This study examines how a specific PSM, the VIPLAN Methodology, has informed the delivery of a research methods course, with the expectation that this improves the students’ ability to problematise a complex situation. Whereas Harwood (2021) explains how the VIPLAN Methodology can be used, this study focuses attention upon a specific application and assesses its impact.

The central research question we ask is ‘what is the impact of embedding a problem structuring approach within a research methods course?’

This question is explored with the delivery of a post-graduate research methods course that was informed by the VIPLAN Methodology. This course was a semester two, ten week long course taken by students on a Master’s Programme in a Business School. It was followed in semester three by the dissertation project which required the students to demonstrate their ability to conduct a research study individually. The study was conducted over three successive years, as well as drew upon data from the preceding two years to provide a reference point.

This is the first study that we are aware of that examines how the principles underpinning a PSM impacts student learning within a research methods course.

It makes two important contributions. The first contribution is empirical and demonstrates to educators that the adoption of an holistic approach to handling PSM can have a real benefit for research methods courses. This has implications for pedagogical practices. The proposal is that there is a shift in course delivery to problem formulation, which allows students to appreciate the challenges of how to establish what the problem is and techniques that assist this (e.g. rich picture building).

This focuses attention upon the second contribution which is conceptual. This draws upon and develops the methodology (VIPLAN Methodology, Espejo 1988) that guides the design and delivery of the research methods course, as initially proposed in Harwood (2016). The methodology offers an holistic approach for handling complex social situations. Complex situations, such as to be found in research into social phenomena, are characterised by the different ways a situation can be perceived by relevant stakeholders (i.e. ‘complexity’, Espejo 1988). Those stakeholders viewed as relevant in defining the question shape the (research) question that emerges, thus drawing attention to the contextual organisational aspects within which problems emerge. Moreover, this approach is not a linear process but iterative, invoking a circular linear process. However, in practice, the process is circular non-linear that moves towards the end state of problem closure. This invokes an approach that supports this, which is the revised version of the VIPLAN Methodology proposed here.

The paper begins by exploring the notion of a problem and explaining how the VIPLAN Methodology assists in handing complex problems. Attention then focuses upon how the VIPLAN Methodology guides the design and delivery of a research methods course. This leads to the development of three hypotheses. There follows an outline of the approach to the study, details about the delivery of the course and then the results of the study. The paper ends with a discussion, which includes the conclusion and implications.

## Dealing with Complex Problem Situations

It is a familiar and significant saying that a problem well put is half-solved (Dewey 1938: 108).

Problems are part of our everyday, including in the world of business. Problems are characterised by situations about which we, individually and collectively feel uneasy. We need to do something. We have a cashflow problem. We cannot retain our employees. Our main product offering has declining sales. Whatever its magnitude, the situation is viewed as problematic (Dewey 1938). We need to resolve our unease. However, the focus is upon solutions and not upon understanding the nature of the problem. Indeed, we might be unaware that there is a lot more to what underpins what we feel unease about – that the situation is complex.

We can approach problems in different ways. One approach is to do nothing in the hope that something happens (‘absolution’) (Ackoff 1994). Second is ‘dissolution’, whereby by changing our understanding of the situation our unease dissolves (Ackoff and Emery 1972). The other two are related and involve doing something within the situation, with, ‘resolution’ producing a satisficing outcome, whilst ‘solution’ achieves the best outcome (Ackoff and Emery 1972). It is proposed that the motivation of the latter two is upon specifying a problem and focusing upon how to solve it rather than having a problem focus, which is to establish what is the problem.

However, problems are not uniform in their make-up, but vary in how their complexity is perceived. If viewed in simple terms, this can lead to situations being unitary defined, well-structured with ‘one best way’ of solving it (Rosenhead 1989). However, there is another class of problematic situations which are challenging to handle due to their recognised ill-structure (Simon and Newell 1958), underpinned by uncertainty and different views about what is the problem (Checkland 1972). The multiplicity of views exacerbates the perceived complexity of the situation (Espejo 1988). This class has been referred to as wicked (Churchman 1967, Rittel and Webber 1973), messy (Ackoff 1974; Eden et al. 1983) and fuzzy (Zadeh 1965; Bellman and Zadeh 1970). These type of situations require the structuring of the situation in order to formulate the problem,. This is likely to involve negotiation amongst the relevant stakeholders to establish what problem to address.

Approaches to the handling of ill-structured problems have emerged in design, for example ‘design thinking’ (Buchanan 1992; Gobble 2014), as well as in operational research, for example Soft Systems Methodology [SSM] (Checkland 1972, 1981, 2000), Strategic Choice Approach [SCA] (Friend and Hickling 1987), Strategic Options Development and Analysis [SODA] (Eden 1988) and the VIPLAN Methodology (Espejo 1988, 1992). These operational research approaches have been labelled Problem Structuring Methods (PSMs) by Rosenhead (1989).

The most prominent PSM is SSM (Munro and Mingers 2002; Mingers and White 2010; Gomes and Schramm 2022). This offers an iterative framework to guide the process by which a solution emerges from a messy situation. It commences with making sense of a situation, in other words, creating a rich picture that provides insight into the complexity of the situation. This enables possible hypothetical solution systems to be defined which are then examined to inform changes to the situation. Its focus is upon the learning associated with the inquiry.

The SSM has informed the development of the VIPLAN Methodology (VM), developed by Espejo (1988, 1992) and examined in Harwood (2021). However, the VM is grounded in second order cybernetics (von Foester 1979). Whilst first order cybernetics focuses upon the object of our attention (the observed system), second order cybernetics is concerned with cybernetics of the observing system. The underlying assumption is that we are all observers, each with our own viewpoint (von Foester 1979), and with the potential to engage in conversations with each other (Pask 1976). The assumption is that the richer our mutual understanding of each others’ views, then it is anticipated that there will be a greater likelihood that complex situations can be better handled. It implies that the relevant stakeholders are involved in this sharing of views. This is consistent with the concept of sensemaking developed by Weick (1988, 1995). Sensemaking is a social and systemic process that involves participants noticing what is going on, bracketing (singling out) and labelling in a manner which organises and makes explicit the different aspects of the situation (Weick et al. 2005). The core concepts within this cybernetics’ perspective are variety and complexity, whereby variety is the number of possible states in a situation and complexity is the number of perceived states (Espejo 1988). The challenge is in how we appropriately handle the variety of the situation through our engagement with its complexity.

**Fig. 1:**
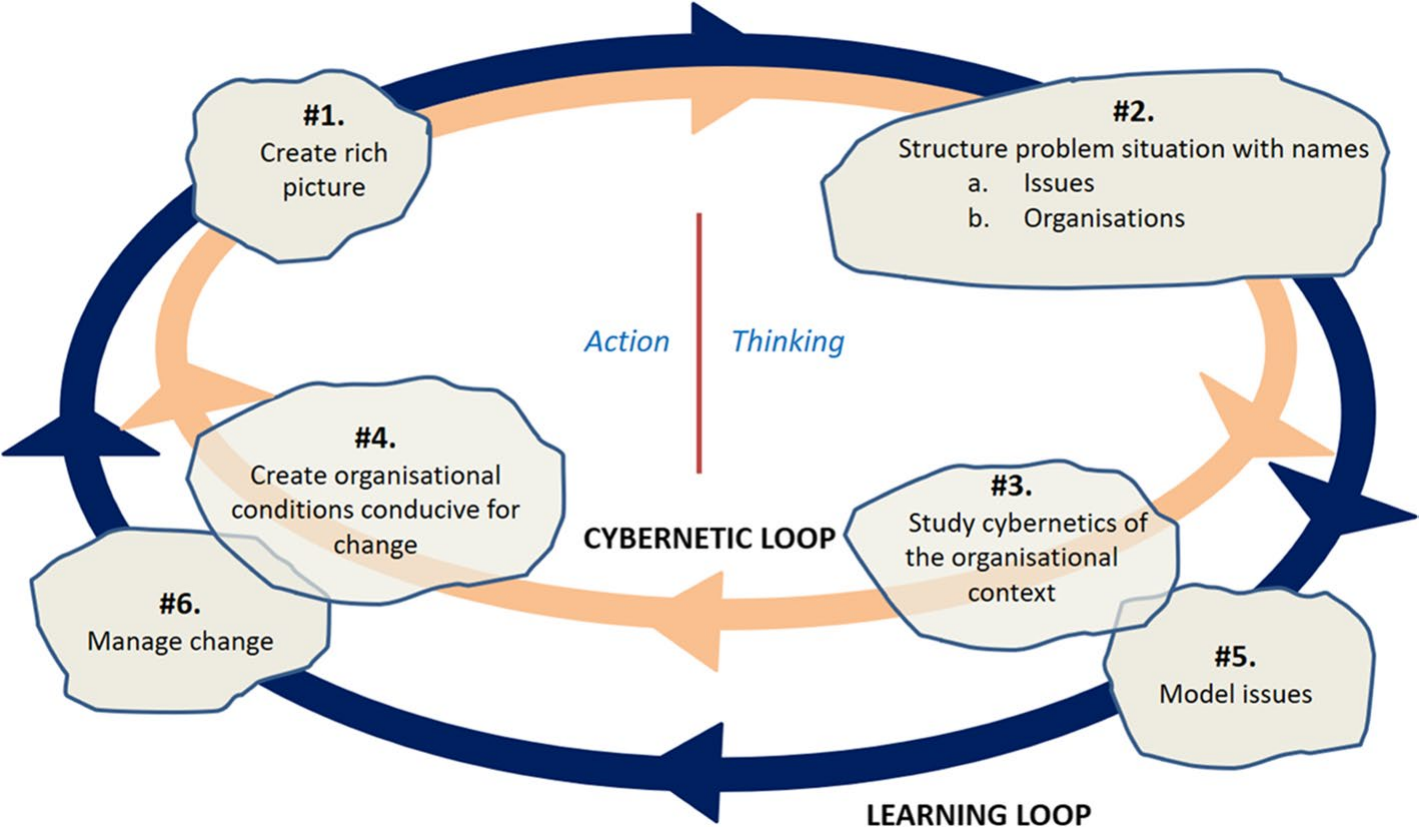
The VIPLAN Methodology (adapted from Espejo 1988, 1992; Bowling and Espejo 1992)

In a similar manner to the SSM, the VM comprises a learning loop (Fig. 1). This involves making sense of situations and reflecting upon insights. It also embraces a cybernetic loop, which is concerned with the organisational contextual conditions (the observing system) that shape the different activities associated with any learning. This focuses attention upon who is included / excluded in any communications regarding the situation and how participants are organised, this shaping any shared insights, negotiations and agreements.

The VM comprise six activities (Fig. 1), these presenting a framework of inquiry that is parsimonious yet sufficient to guide the handing of a problem situation.

- Activity 1 (#1) is about understanding the situation in order to develop a rich appreciation of the different views about what constitutes the situation. This creation of a ‘rich picture’ (cf. Checkland 1972, 1981) may involve mapping out the different elements in informal sketches, though there are no rules about how this is done (Lewis 1992; Bronte-Stewart 1999) and can be merely mental images (Bronte-Stewart 1999). Bell et al. (2019) draw attention to the challenges of interpreting and analysing rich pictures.
- Activity 2 (#2) is the act of focusing in upon the question to be addressed. The structuring of the situation through the question allows the boundaries of the system in focus to be defined and the relevant stakeholders identified. This can be an iterative process since those involved in conversations can reshape the question that emerges.

Activities 3 (#3) and 4 (#4) relate specifically to the cybernetic domain.

- Activity 3 examines the organisational requirements to enhance the likelihood that the requisite conversations in the learning loop can take place. This establishes who needs to be involved and how they are organised. The Viable System Model (VSM) (Beer 1979, 1985; Espejo and Harnden 1989) offers a organisational diagnostic and design device.
- Activity 4 is about implementing the insights from #3.

Activities 5 (#5) and 6 (#6) relate specifically to the learning domain.

- Activity 5 is about analysing the issues raised by the question, which can involve modelling (e.g. simulation, prototyping).
- Activity 6 is about implementing the insights from #5.

**Fig. 2:**
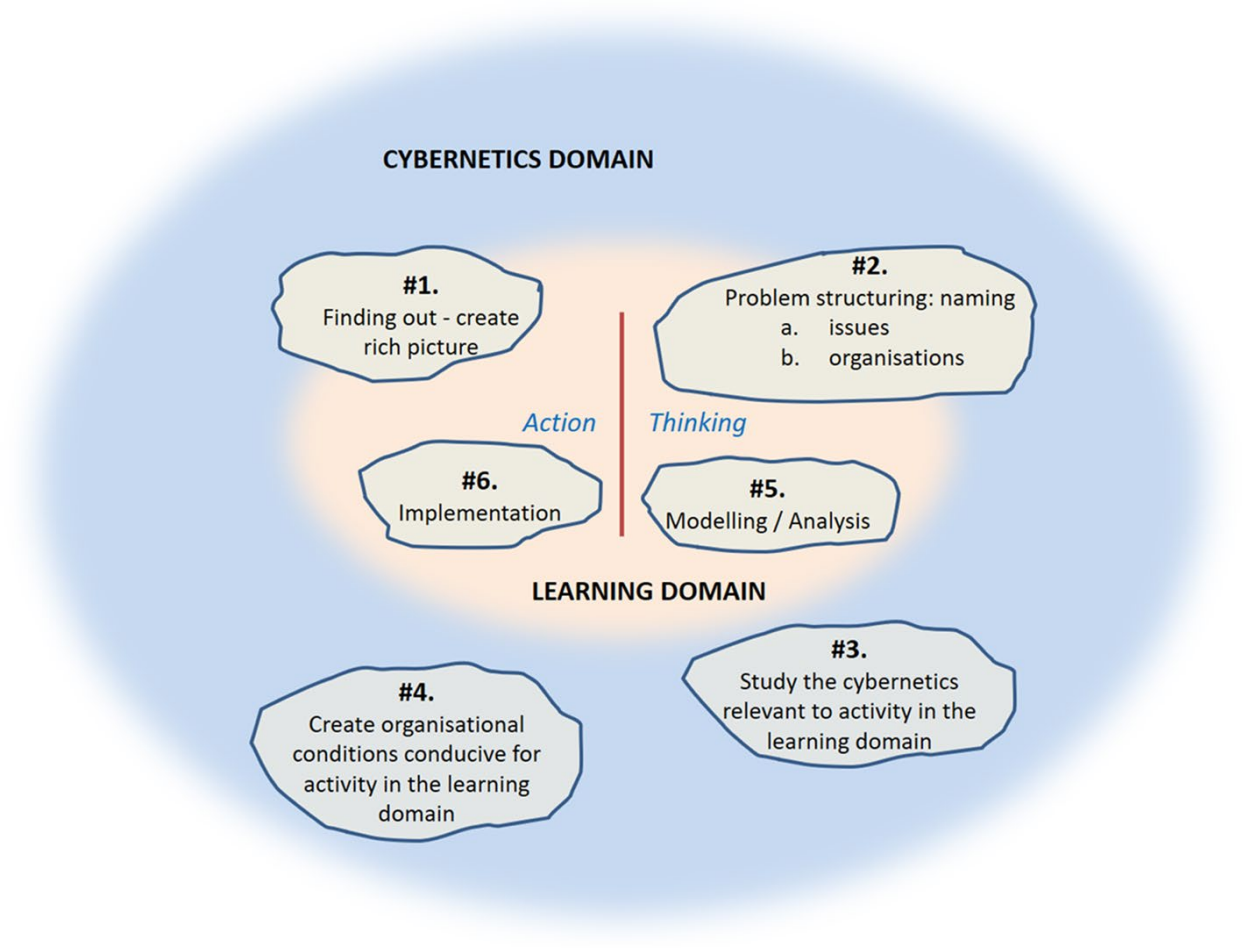
The VIPLAN Methodology (revised)

However, the author’s experience of using the VM to handle complex situations revealed that the arrows and loops were implying a prescribed circular linearity which was potentially confusing. Instead, as proposed, in Harwood (2021), if the activities act as ‘pointers’ that guide both thinking and action, then there is no prescribed use of these pointers. Instead, an iterative use of the model is a circular non-linear process. This suggests that when we think about the content of our inquiry (learning), then there is likely to be a need to consider whether the organisational (cybernetics) aspects are conducive to this learning and associated action. If not, then attention focuses upon improving the conduciveness of these conditions. Thus, the relationship of the activities related to the interplay between two domains, the cybernetics domain and the learning domain, arises at any stage of dealing with the problem situation. A revision to Fig. 1 to reflect this is presented in Fig. 2.

This presents #1 and #2 as spanning both domains. This recognises that #1 is about understanding both the issues raised that relate to the situation and also the relevant stakeholders and how they are organised. Likewise, #2 is about structuring the problem recognising the distinction between the problem and the organisation of the stakeholders who are associated with the named problem. For the other two activities (#5, #6) in the learning domain then the question is whether the organisational conditions are conducive to these, invoking activities #3 and #4 in the cybernetics domain. Whilst the methodology does not preclude intuition, it focuses attention upon analytical reasoning (Mohaghegh and Größler 2020).

**Fig. 3:**
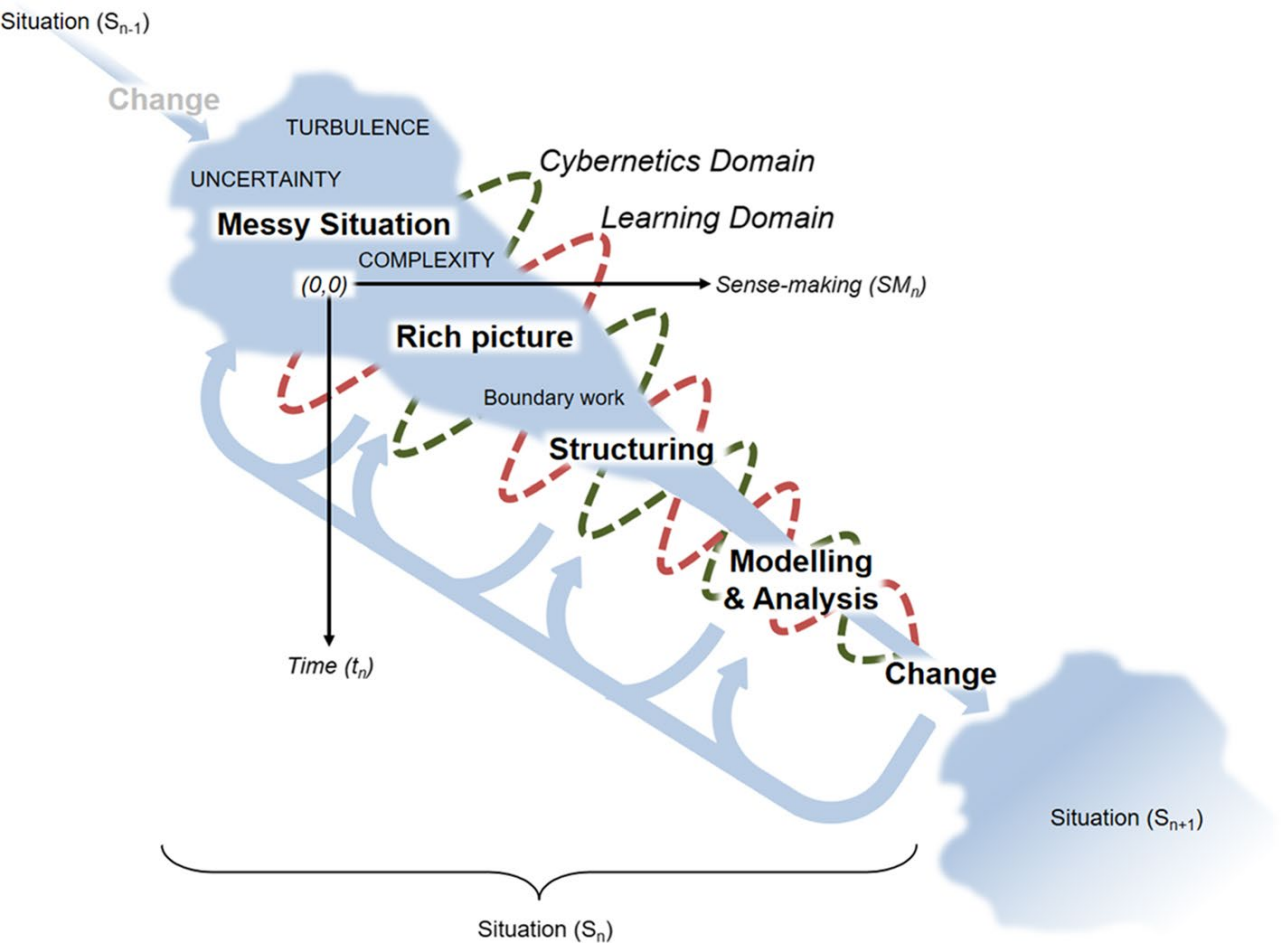
Dealing with a problematic situation using the VIPLAN Methodology

The overall process of inquiry is one which shifts *from* a messy situation characterised by complexity, uncertainty and turbulence, with vague boundaries about what might or ought to be considered *to* a clearly bounded and focused state which results in some form of change. This change may be a deeper understanding of the situation or change to the situation. This is captured in the ice-cream cone model (Fig. 3).

In simple terms, the challenge is to explore the situation in such a way as to identify and organise its many features and make meaningful sense of it (rich picture [#1)). With this rich understanding, the aim is to establish what to focus upon [#2], this perhaps involving negotiation with relevant stakeholders. The boundaries of the situation become increasingly more firm with clearer definition and focus of the question. Ongoing engagement within the situation, is likely to involve an iterative process of modelling possible solutions [#3, #5] to guide actions [#4, #6], with perhaps redefinition of the investigated question. Further, for every situation handled, others will emerge to require attention.

The value of this reinterpretation is that students can develop a more holistic appreciation of the focused handling of complex situations and its challenges, especially relating to question formulation, within the tight time constraints of course delivery, without the necessity of penetrating the technical aspects of the methodology.

## Dealing with Complex Problem Situations in an Educational Setting

Within an educational setting, courses have tended to handle problems by solving a large number of problems (Taconis et al. 2001). However, in solving a problem, Schoenfeld (1988) makes the distinction between rote memorization of the procedure to deal with the problem and understanding why the procedure works. The former can be associated with the view that the lecturer transmits content to be absorbed by the student (Papert 1994) and converts into superficial learning (Entwistle and Peterson 2004). The latter view can be associated with the notion that material is presented to the student, then is interpreted and constructed by the student (Papert 1994; Martin 2007), this encouraging deep learning, understanding and the ability for independent thinking (Entwistle and Peterson 2004).

This latter focus upon understanding invokes the value of application and underpins the case method, or Socratic Method, introduced into Harvard Law School in 1870 (Moskovitz 1992). The case method, through its dissection and discussion, would reveal how the law worked (i.e. the process), in contrast to the passive activity of reading and listening to lectures. This supported the practical need for lawyers to solve problems. However, the case method appears to be grounded in a situation and / or problem that is defined, with emphasis upon solution. It is unclear how the situation is constructed and how the problem is established. Indeed, how to make sense of the situation in order to establish the research question is one of the challenges facing social science researchers, including dissertation students. This invites the question of how problem formulation is introduced into a research methods course in such a way as to develop a deep understanding of what this involves.

## Complex Problem Situations and a Research Methods Course

This reasoning has specific relevance to research methods courses, which are delivered in many disciplines (Wagner et al. 2011). However, studies have revealed that research methods is one of the most challenging courses to deliver, with student engagement poor (Denham 1997; Murtonen and Lehtinen 2005; Earley 2014). Takata and Leiting (1987) state that the traditional lecture mode of delivery involves students to passively receive descriptive accounts. Moreover, Earley (2014) reveals that students are unreceptive to research methods courses for reasons that include perceived lack of relevance, anxiety about perceived difficulty of the course, disinterest, poor attitude towards research and misconceptions about research.

Consequently, efforts to overcome these challenges have shifted from passive delivery to experiential learning-by-doing approaches (Schutt et al. 1984) and include learning approaches that are active (reflective), problem-based, co-operative (shared), service (community based) and experiential (‘by doing’) (Earley 2014). This practical orientation can improve perceived relevance and understanding of research. However, as commented upon by Strangman and Knowles (2012) about research methods courses and Bhardwaj et al. (2018) about strategy courses, the emphasis is upon problem solving rather than problem formulation, and specifically, how to arrive at the question.

A shift in emphasis from problem solving to include problem formulation allows research to be viewed as a form of inquiry into a problematic situation. Further, if students are regarded as future consumers of research, as well as future active participants in problem-solving (Good 1936; Schutt et al. 1984; Hardcastle and Bisman 2003) then the potential benefit is to improve the student’s general skills of inquiry (Schutt et al. 1984: 235–236) which may enhance the student’s appreciation of the value of a research methods course. However, it is unclear how problem formulation is to be accommodated in a research methods course.

One approach is presented by Strangman and Knowles (2012) who demonstrate how research questions can emerge from brainstorming and related activities in class based group-work. Whilst this places emphasis on activities, this reveals the opportunity to develop a student’s understanding of the nature of messy situations and more general approaches to handling complex situations.

Whilst it is within the specialised domain of PSMs that the attention focuses upon problem formulation, the delivery of courses about PSMs is problematic (Ackermann 2011; Ackermann et al. 2020). Challenges of delivery include student discomfort with the notion that situations are characterised by complexity and uncertainty, that there is not a ‘right’ answer, as well as the difficulty in creating an appropriate opportunity to experience the implementation of a PSM.

This leads to the conclusion that, whilst problem orientated courses enhance the student learning experience, these tend to emphasise problem solving rather than problem definition. Further, courses that explicitly deliver problem structuring are problematic for students due to the mindset demands. Moreover, when students are expected to complete an undergraduate or postgraduate dissertation that demonstrates their ability to conduct an independent research study, amongst the challenges they face, is that of determining a focused research question for their study (Shaw and le Roux 2017). This raises the challenge of whether it is possible to deliver a research methods course that embeds the principles of problem structuring so that students are better focused upon their dissertation topic.

### Application to a Research Methods Course

This paper examines the tacit integration of the principles of a PSM within a research methods course and its impact upon the subsequent dissertation.

**Fig. 4:**
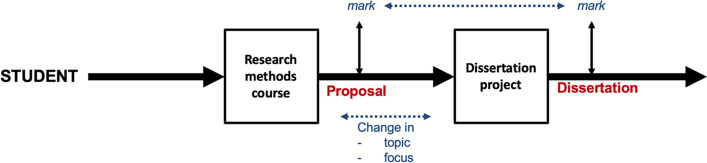
The student learning experience of developing research competence which is ideally applicable to experience after the post-graduate programme

The occasion to deliver a research methods course prior to the dissertation phase of the postgraduate programme provided the opportunity to evaluate whether approaching a research methods course as a form of complex problem handling (this is referred to as a ‘problem oriented learning strategy’) and teaching the principles and challenges of finding a research question, enhanced engagement with the course. Due to the constraint of time, the emphasis was to explain why situations are messy and difficult to solve and the principles and pragmatics of how to handle these complex problems rather than the VIPLAN Methodology itself. Upon completion of the course, students were to submit a research proposal addressing a focused research question that they selected themselves (Fig. 4). Further, it was anticipated that this proposal would then be investigated in the subsequent dissertation project, which was a separate course. The dissertation was a project of around three months that was intended to assess the student’s ability to conduct an independent research project.

The expectation was that better engagement with the course would lead to a higher mark in the proposal. Moreover, that whatever the performance was for the proposal, there would be a corresponding performance in the dissertation. Finally, that with an appreciation of how to establish the research question, then there would be less likelihood of a change in topic or focus during the dissertation project. This leads to three hypotheses.

The first hypothesis relates to student engagement with the course. If the cohort were better engaged with the course then it might be expected that they performed to a higher standard than in previous years, this being reflected in a higher average mark for the assessment:

- H1: Better student engagement with the course will result in a higher average mark for the assessment of the proposal in comparison to previous years.

The second hypothesis concerns the transfer of learning from the research methods course into the dissertation process. A better, more holistic, understanding of what is involved in research is one that seeks out a meaningful topic to study, formulates the research question, then applies an appropriate research design to complete the study. Thus, it is proposed that a more holistic approach, which includes problem formulation, is likely to manifest in the marks/grades earned for both the research proposal and the subsequent dissertation. It is assumed that students will engage with the course materials for the purpose of understanding how to improve their approach to their research proposal and hence their performance in the dissertation. Consequently, we can expect that students’ learning from the course will feed into the dissertation process. Thus, if effective, there should be a strong correlation between marks/grades earned for research proposals and dissertations. The underlying assumption is that a student’s performance is relatively consistent across courses, especially if there is complementarity between courses. If a student’s mark is improved, this may be due to better quality of dissertation supervision. It would not be expected that a student has a lower dissertation mark:

- H2: A problem oriented learning strategy is associated with a correlation between marks/grades for research proposals and subsequent dissertations.

The third hypothesis concerns changes in the dissertation topic, with fewer changes in the dissertation topic being proposed as an indicator of student understanding of how to select a research question due to the problem oriented learning strategy of the course:

- H3: A problem oriented learning strategy is associated with earlier identification of a stable dissertation topic with less likelihood of a change in topic during dissertation phase.

## Approach to the Study

This exploratory study is set within the context of a research methods course delivered over a three year period to MSc students in Management in a UK University. The researcher was also the person responsible for the course including its delivery. As such, this is primarily an ethnographic critical reflection, carried out in adherence to the interpretivist tradition, but recognising the commensurability of mixing qualitative and quantitative methods (Bryman 1984; Hanson 2008; Harwood 2011). To provide a frame of reference for the delivery period, data was drawn from records of the previous two years. Thus, this offers a five year longitudinal case study.

### Background and Developments to the Course

This was an established second semester postgraduate course. Its objectives were to develop the student’s understanding of research methods, particularly in the context of business, and specifically, to develop the student’s ability to accomplish a meaningful piece of independent research for a Master’s dissertation. It had been taught by different people over previous years, mainly in a traditional lecture format. The size of the class cohort varied between 50 and 70 students per year. It comprised an international cohort with students coming from different learning cultures, for example, around half of the students were from East and South East Asia, whilst 30–45% arrived from Europe (including UK).

The course was delivered in two hour blocks over ten weeks, with five additional two hour blocks for delivery of quantitative material. Further, non-compulsory sessions were provided that introduced students to such tools as SPSS. Assessment was based on a research proposal, which preceded and was expected to inform the research dissertation project (Fig. 4). Supervisors were allocated after the research proposal has been submitted, which could lead to a change in the dissertation topic following student-supervisor discussion.

Over the three years there were a number of developments.

#### Year 1

A review of previous delivery of the course revealed a more traditional approach to delivery, with material about the process of how to find a research question forming only one part of one session. In response, the first session of the ten sessions was orientated towards understanding the nature of messy situations. This introduced the notion of a rich picture and the challenges of establishing a research question, these including the limitations of what was possible to address as well as the issue of significance based on Huff 2008. This theme ran through the subsequent sessions as appropriate.

A few days prior to the first session, students were asked to think about possible topics for their dissertation. These were intended be used by students as material for in-class exercises aimed to support their understanding of the topic of the day. Further, it would contribute to the development of their research proposal. Within a few weeks, feedback revealed that some students were feeling under pressure about identifying appropriate topics, exposing a division between those who had a topic and those who were struggling to find one. Additional feedback sought during a subsequent session, revealed other issues such as difficulty with concepts (cf. Murtonen 2015) and the desire for stronger emphasis upon practical examples. An extra session was then introduced that week to help students still struggling to find a project topic.

#### Year 2

Based on feedback and reflection about the first year delivery, there were three significant changes. First, was the move of the first lecture to early November of the previous semester to give students time to seek out possible projects for the start of the second semester course. Second, was the introduction of an assessed reflective log to enable students to reflect over, as part of their learning experience, how they were developing their research proposal (Güss and Wiley 2007). This aimed to reveal student concerns whilst developing their proposals. Third, content was modified to give greater attention in earlier sessions to developing student appreciation of situational complexity and the use of the rich picture to draw out this complexity.

#### Year 3

Again based on feedback and reflection about the previous year’s delivery, there were two relatively significant changes. The first involved a reduction in the number of mainstream lectures, with the latter two replaced with drop-in sessions to help students with any issues they had relating to the development of their proposals. The second was the collection of feedback about student expectations regarding the course at the beginning of the first session. This was to identify gaps (or is it misunderstandings?) between expectations and learning outcomes and clarify these at the beginning of the second session.

### Data Collection

The data comprises three forms. First, are research proposals submitted at the end of the research methods course and submitted dissertations. Second, are the marks awarded to both research proposal and dissertation. Third, to provide an insight into the student experience of the research course, three sources were used:

- informal, anonymous course feedback collected from students in-class towards the middle and end of the course in the first and second years and towards the middle and at the start of the course in the third year, this latter feedback revealing preliminary student expectations. The change in the third year to drop-in sessions prevented the collection of end of the course feedback.
- formal, anonymous end-of-course feedback used to collect information about courses, and allowing non-attendees to respond.
- anonymised assessed reflective log, which was part of the submitted assessment. One aim was to establish how the students were engaging with the course and the process of producing a research proposal. Submissions varied considerably in terms of content, ranging from basic descriptive accounts of lectures to more refined offerings comprising brief descriptive accounts of activities and decisions, explanation of rationale and underlying thoughts and feelings as well as the value of materials to future careers.

Due to low and variable response rates of anonymous feedback, the emphasis was upon the qualitative insights rather than their quantitative profiles. Quotes are presented using the following convention: year (e.g. yr2) is followed by an identifier to identify source (e.g. a number for log; ‘F’ to designate formal feedback; ‘I’ to designate informal feedback).

## Analysis and Findings

The insights of this study suggest that there was a positive impact of this problem formulation orientation. Not only did student expectations reveal question definition as an issue, but the analysis of student marks and the change in topic-focus suggested that this more holistic approach to course delivery addressed this specific expectation as well as had a more general positive impact. This is further examined in the following evaluation of these expectations and the three hypotheses.

### Student Expectations

Student expectations reveals what students anticipated to gain from the course. Collected at the start of the third year, an analysis of student expectations revealed the importance of five specific themes that accounted collectively for a significant proportion (81%) of responses: learning about methods (43%), how to produce a dissertation (36%), how to establish research questions (31%), how to do research (26%) and, lastly, how to write academically (20%). There was also concern about how to produce a literature review (11%) and a research proposal (11%), with a few raising the issues of critical thinking, lifetime benefit and time management. One student did not know what to expect.

This suggests practical concerns. The emphasis is upon the subsequent dissertation and how to get there in terms of establishing the topic (research questions) and methods to use, with the literature review and style of presentation (write up) being concerns. The research proposal appears important insofar as it is assessed as well as feeds into the dissertation. Just under a third of the students revealed research questions as an expectation.

### Hypothesis 1

The first hypothesis was that the level of student engagement could be established by the overall performance of the cohort, this manifesting in a higher average mark for the assessment in comparison to previous years. The average and standard deviation for each year is presented in Table 1. This reveals that the average did rise in years 2 and 3, but that this was only by one to two points. The standard deviation rose to around 10 for the second and third years then fell to around 7 for the latter two years.

**Table 1:** Average mark and standard deviation for proposal assessment

	Previous deliveries		Personally delivered		
	Year − 2	Year − 1	Year 1	Year 2	Year 3
Average (Ave)	64.7	64.4	62.5	66.8	65.3
Standard Deviation (SD)	8.8	10.4	10.2	7.2	7.6

This is open to interpretation. There are two factors that can aid interpretation. First, marking is to the university’s ‘common marking scheme’, which should enable consistency in how marking is conducted irrespective of which year. Second, the standard will be set by what students are told regarding marking criteria, which were refined over the three years of delivery to reflect increased focus upon problem formulation and coherence. Whilst the expectations may change from year to year, the consistency enabled by the ‘common marking scheme’ ought to permit a year to year comparison. This suggests that the averages and standard deviations indicate that there was better student engagement in the latter two years.

### Hypothesis 2

In contrast, if marking is viewed as relative to within a year, then the second hypothesis has validity, which focuses upon an individual student’s performance and the correlation between one course assessment (proposal) and a subsequent course assessment (dissertation). The degree of co-variation between the research proposal mark (independent variable) and the dissertation mark (dependent variable) was assessed using the Pearson cross-correlation, since it is an appropriate technique for interval variables requiring bivariate analysis. This analysis (Table 2) reveals an overall improvement in this association from year-2 to year 3, with a leap between year 1 and year 2.

**Table 2:** Correlations between proposal marks/grades and dissertation marks/grades over the period of investigation

	Previous deliveries		Personally delivered		
	Year − 2	Year − 1	Year 1	Year 2	Year 3
Cross Correlation coefficient	0.303	0.355	0.391	0.511	0.531
*P* value	0.014	0.006	0.003	< 0.0001	< 0.0001
Mark difference (10 or more)	26%	29%	45%	20%	21%
Percentage of marks increased	53%	53%	80%	42%	80%

Year-2 was the first year of course delivery for the previous lecturer, with the learning curve resulting in a better outcome the following year. By drawing upon the lessons from previous years and orientating the course to problem solving, year 1 saw yet another small improvement in the correlation. However, the changes of delivering the first lecture earlier and the introduction of project log appear to have been responsible for the leap in year 3, though it is unclear their respective impacts. The earlier timing provided time to consider possible projects. However, the log had mixed reception, with some students expressing appreciation of it in their log:

Keeping a log like this helped me know my progress and get my thoughts clear. I should keep this habit for my dissertation (yr2-58).

In contrast, there were two comments in end-of-course feedback revealing negative views: “reflective log useless” (yr3-F), “Remove the Log from the course. It is unnecessary and not helpful at all” (yr2-F).

A complementary analysis examined whether a person’s dissertation mark rose or fell relative to the proposal mark (Table 2). An examination of mark differences between proposal and dissertation of ten or more (i.e. a band shift) revealed a consistent pattern across all five years, with only a few exceptions. There was less of a mark difference in years 2 and 3 compared to previous years, though the percentage of marks that increased within a cohort was 42% and 80% respectively (Table 2). Within this subset, lower proposal marks were associated with higher dissertation marks, whilst higher proposal marks were associated with lower dissertation marks. Further, proposal marks in the 30s were significantly raised by a minimum of 24 with a 35 proposal mark having a dissertation mark of 70 awarded.

Overall, this suggests that over the five years, the proposal mark was becoming a better determinant of the dissertation mark. Whilst the impact of the supervisor upon the dissertation mark is unclear it is perhaps revealed in the significant rise of the dissertation mark over the proposal mark. Nevertheless, in view of all the factors that can impact performance in the dissertation, the research methods course appears *not* to be insignificant.

### Hypothesis 3

The third hypothesis concerned changes to the topic and focus in the dissertation from that stated in the proposal. A comparison was made between the title and aims stated in the proposal and the dissertation title and abstract. The independent variables are the proposal topics and themes. Each pairing was categorised as ‘focus change’, ‘topic change’ or ‘no change’ based upon the terminology used. A topic is defined as the subject matter or theme of interest (e.g. tourism, e-commerce), thus a change in topic is to move to another subject. Focus is the specific application or theme. A change in focus is a shift in application or theme within a topic. For example, within the topic of ‘strategic competition’, the shift in focus is from ‘open innovation’ to ‘patents’. Where there was ambiguity then the main text of both were examined for clarification. The categories were expressed as a percentage of the total number of pairings for each year (Table 3).

**Table 3:** Changes in dissertation topic and focus over the period of investigation

	Previous deliveries		Personally delivered		
	Year − 2	Year − 1	Year 1	Year 2	Year 3
Focus changed	14%	7%	4%	10%	2%
Topic changed	6%	7%	14%	5%	6%
Total change	20%	14%	18%	15%	8%

It was assumed that, if the problem oriented approach introduced into the research methods course was successful, then it could be expected that students would be less likely to change their topic or focus. The analysis (Table 3) revealed an unexpected result. In the two preceding years, student focus on their topics had improved, whilst changes in the topic itself were insignificant. In contrast, in year 1 of delivery there was a relatively high number of topic changes. In year 2, which had the earlier first lecture and log, the number of topic changes dropped, whilst the change in focus increased. This might be expected if students had a better idea of what they were doing as a result of having more time to explore their topic. The third year of delivery, with improvements in delivery based upon the experience of the previous two years, resulted in fewer changes in both topic and focus, this supporting the assumption stated at the beginning of this paragraph. It is proposed that, whilst it is to be expected that there will still be students wanting to make changes for a variety of reasons (e.g. data access, contribution, supervisor advice), by placing emphasis upon finding the right project at the proposal stage, then the student is less likely to change the topic or focus of the dissertation.

The emphasis upon student understanding of the principles of what was involved in establishing a focused topic addressed the expectation of the third of students who required the course to help them establish research questions. Whilst this better understanding appeared to enable the student to be more effective in establishing their research question, this better understanding of what was involved did not necessarily alleviate the challenge of the experience, as revealed in the feedback and logs. The effort required can be initially underestimated:

“I never imagined how challenging the decision of the topic of the project could be” (yr2-48).

“development of the dissertation’s topic itself became a lengthy process, exceeding initial expectations” (yr3-51).

The emergence of topics could be both unexpected and on-going:

With the deepening of theoretical study and practice, I continually encountered many new problems, all of which were not expected (yr2-61)

Likewise, efforts to make notes about interesting topics “became really messy and after a short while and my notes were all over the place” (3 year-21). For some, this can involve quite an extensive search process, drawing upon a variety of sources such as past dissertations, academic literatures, media, contacts and potential dissertation supervisors. Further, reading extensively need not help: “my idea is messy after doing lots of reading” (yr3-22). One student who attempted to focus upon one potential supervisor’s topic realised that this was not the way to do it, as this was “restricting myself” (yr3-32). A few logs revealed the value of presenting research as a form of complex problem solving as it prompted such approaches as “I started to ask myself questions…” (yr2-41). For some, one criterion was finding a topic aligned with future work aspirations. Additionally, serendipity had a role: “a number of incidents happened which opened my mind and brought me close to my topic” (yr2-44). Irrespective, the logs revealed the use of emotive words to explain aspects of this process such as ‘worry’, ‘doubt’, ‘hesitation’, ‘stress levels’, ‘panicked’, ‘puzzled’, ‘intimidating’, ‘confusing’, ’clueless’, ‘very lost’, ‘overwhelming’, ‘bothering’, ‘frustrating’, ‘struggle’ and ‘disappointment’, as well as ‘rewarding’, ‘comfortable’, ‘great feeling’ and ‘happy and relieved’. This highlights the potentially emotive nature of the challenge in finding an appropriate project; “is still a miserable experience for me to explore dissertation topics” (yr2-53). There was little evidence of enjoyment.

## Summation and Conclusion

### Overview of Study

This exploratory study examines the impact of embedding the principles of problem structuring into a research methods course. This responds to the call from Bhardwaj et al. (2018) and Strangman and Knowles (2012) for attention to be given to problem formulation within university courses. It draws upon the principles of a PSM, the VIPLAN Methodology. This methodology guides the handling of complex situations from awareness of the need to do something to ‘closure’. This resulted in a change to the delivered research method course from one that focuses on the more traditional content of philosophical underpinnings, design and techniques with emphasis upon problem solving, to one that is more holistic by considering research as the handling of a complex situation with the need to generate meaningful insights. Problem formulation was introduced at the start of the course, and the students were encourage to reflect upon their learning experience through the log. Despite the limitation of the short duration of the study, nevertheless, it suggests that the problem orientation introduced into the research methods course did have a positive impact.

### Key Findings

The three hypotheses aimed to explore this holistic approach to a research methods course by examining its impact upon a follow-up dissertation project. Each of the three hypotheses appeared to be supported. The course average and standard deviation suggested that student engagement with the course was improved. The correlation between marks/grades for research proposals and subsequent dissertations suggested that the proposal was becoming a better determinant of the dissertation mark. The comparison between proposal and dissertation suggested that students appeared to be less likely to change their topic or focus.

Whilst not explicitly examined in this study, a plausible explanation for this impact can be explained with the conceptualisation of sensemaking as presented in Weick et al. (2005). The process of getting to a research question for the proposal and dissertation, which relates to activities #1 and #2 of the VIPLAN Methodology, is the action of sensemaking. However, rather than this being for collective action, this is sensemaking to support individual activity, though this may draw upon the insights of others. As a process, the ‘search for meanings’ involves the noticing and the bracketing of topics. It is an organising and filtering activity. However, this is not a linear process, but can be an emotional struggle, invoking an emerging and unpredictable path, fusing the search for a meaningful topic that is feasible as a research project within the time constraints and a sense of self-fulfilment. What emerges as the research question is something that resonates with the individual’s sense of self. The outcome is that the research proposal is a more feasible, suitable and acceptable proposition for the dissertation, with there being less likelihood of change in topic or focus.

### Contributions and Implications

This study makes two important contributions.

The first is conceptual. This presents a revised interpretation of the VIPLAN Methodology (Espejo 1988), this viewing the handling of complex social situations holistically as a circular non-linear process, rather than the iteration of a linear process (circular linearity). This reflects the unpredictable nature of how a complex situation tends to be handled, with any point in its handling leading to an appraisal of what to do next. The revised interpretation of the VIPLAN Methodology makes the distinction between the cybernetics domain and the learning domain, the former shaping the latter, which is potentially relevant at any point in the process. This duality is potentially present in all forms of inquiry. Further, this interpretation enhances a non-technical articulation of the principles of the VIPLAN Methodology, since it eliminates the implied causality indicated with the arrows of its original presentation (Fig. 1). This translates into greater accessibility to the principles of the interplay of the cybernetics and learning domains and how they mutually shape each other. To add is the use of Weick’s sensemaking to explain activities #1 and #2 of the VIPLAN Methodology, this usefully contributing to our understanding of how we get to the question being addressed.

The second is empirical and demonstrates to educators that the adoption of an holistic approach to handling complex situations can have a real benefit for research methods courses. It spotlights the complexity, uncertainty and turbulence of situations and explains how these can be handled in such a way that assists students handle the challenge of establishing their research question. This is particularly relevant for students with mind-sets that are used to certainty and predictability (Ackermann 2011; Ackermann et al. 2020). Whilst it did not eliminate the challenging nature of finding the research question, the introduction of such techniques as rich picture building and stakeholder analysis provided students with methods that could aid their search and sensemaking. Moreover, students who had a good appreciation of the process of problem formulation for the proposal would be less likely to change their topic or focus in the dissertation stage. This suggests that for business courses where there is an aspect of the course that involves making sense of complex situations and problem definition, then incorporating insights that address activities #1 and #2 can improve student appreciation of *how* to make sense of these situations.

### Conclusion

Research is interpreted here as a form of inquiry into complex situations, which involves the process of not only of solving a problem, but also establishing what the problem is (problem formulation). This spotlights the complexity, uncertainty and turbulence of situations and explains how these can be handled. This invites the VIPLAN Methodology. This recognises the circular non-linear nature of research and offers an approach for handling complex situations. It comprises of six activities which act as pointers within the inquiry process. Moreover, it makes the conceptual distinction between the learning domain and the cybernetic domain, this duality potentially existing for all forms of inquiry, therefore with its attention leading to more appropriate responses to situations. Consequently, it is appropriate to incorporate the principles of the VIPLAN Methodology into a research methods course and thus improve the student understanding of complex situations, their challenges and also how to be more effective in dealing with them. This understanding is expected to be demonstrated in the assessment of a research methods course as well as any follow-up dissertation project.

This exploratory study, which sought to explore the validity of this argument, indicates improved student engagement with the course which leads to the more effective selection of a dissertation topic. In other words, it aids problem formulation and the establishing appropriate questions. This is important as research requires the appropriate formulation of problems if it is to be focused and meaningful. Moreover, it is expected that this understanding has broader implication and responds to the World Economic Forum’s (2016, 2018) call for students to develop the ability to handle complex problems for the workplace. Thus, the proposed new approach to a research methods course is expected to provide students with relevance well beyond their period of study.

## Data Availability

Research data is not available to preserve privacy.

## Declarations

### Employment

Am employed by the University of Edinburgh.
